# Spectrum of tumors in the families with Hereditary Breast Ovarian Cancer syndrome carrying germline mutations in BRCA1 and 2 genes

**DOI:** 10.1186/1897-4287-10-S1-A2

**Published:** 2012-01-12

**Authors:** M Budryk, J Pamuła-Piłat, K Tęcza, E Grzybowska

**Affiliations:** 1 Genetic Counseling In Outpatient Clinics, Maria Skłodowska-Curie Memorial Cancer Center and Institute of Oncology, Gliwice Branch, Poland; 2 Center for Translational Research and Molecular Biology of Cancer, Maria Skłodowska-Curie Memorial Cancer Center and Institute of Oncology, Gliwice Branch, Poland

## 

The aim of genetic tests performed by Genetic Counseling at Centre of Oncology was to screen for carriers of germline mutations in BRCA1 and 2 genes who are at high risk of developing breast or/and ovarian cancer.

Among the patients qualified to genetic tests there are the patients who do not meet the Amsterdam criteria of HBOC syndrome.

In Genetic Counseling at Centre of Oncology we find germline mutations in BRCA1 and 2 genes in about 5% of patients. Beginning in January 2003 until now we found mutations in BRCA genes in 247 families. Not all families belong to the group of high risk of developing breast or ovarian cancer. Among them there were 23 families which did not meet pedigree and clinical criteria.

Analysis of tumors which were developed by the members of families carrying germline mutations in BRCA1/2 genes revealed mainly breast (415) and ovarian (170) cancers. There were also tumors which were not typical for this mutation. The most frequent not typical tumors in the families with germline BRCA1/2 mutations were: lung cancer (38), intestinal tract tumors (26) – including stomach cancer, liver cancer (20), pancreas cancer (15) and also hematopoietic tumors – among them leukemias (17), prostate cancer (14) and other.

These findings justify performing genetic tests for germline mutations in BRCA1/2 genes not only for the patients who meet pedigree and clinical criteria typical for HBOC syndrome.

